# Trends in Radiation Exposure With the Refinement of Radiation Exposure Categories in Congenital Cardiac Catheterization: Insights From the CRISP Registry

**DOI:** 10.1016/j.jscai.2025.103727

**Published:** 2025-07-23

**Authors:** Gurumurthy Hiremath, Juan Carlos Samayoa, Alexander Javois, Osamah Aldoss, Ryan Leahy, Reid Chamberlain, Elena Amin, Marko Vezmar, Makram Ebeid, Shyam Sathanandam, David Nykanen, Thomas Forbes, Christopher Curzon, Martin Bocks, Daisuke Kobayashi

**Affiliations:** a Division of Pediatric Cardiology, Department of Pediatrics, Masonic Children's Hospital, University of Minnesota, Minneapolis, Minnesota; b Section of Pediatric Cardiology, Advocate Children's Hospital, University of Illinois Hospital, Chicago, Illinois; c Division of Pediatric Cardiology, University of Iowa Stead Family Children's Hospital, Iowa City, Iowa; d Department of Cardiology, Children's Hospital of Colorado, University of Colorado, Aurora, Colorado; e Division of Pediatric Cardiology, Department of Pediatrics, Duke University Medical Center, Durham, North Carolina; f Division of Pediatric Cardiology, UCSF Benioff Children's Hospitals, University of California, San Francisco, San Francisco, California; g Children's Minnesota, Minneapolis, Minnesota; h Department of Pediatric Cardiology, University of Mississippi Medical Center, Jackson, Mississippi; i Division of Pediatric Cardiology, Department of Pediatrics, Le Bonheur Children's Hospital, University of Tennessee Health Science Center, Memphis, Tennessee; j Division of Cardiology, Arnold Palmer Hospital, Orlando, Florida; k Division of Cardiology, Joe DiMaggio Children's Hospital, Hollywood, Florida; l Division of Cardiology, Children's Nebraska, Omaha, Nebraska; m Division of Cardiology, Case Western Reserve University, University Hospitals, Cleveland, Ohio; n Division of Cardiology, Department of Pediatrics, St. Louis Children's Hospital/Washington University School of Medicine, St. Louis, Missouri

**Keywords:** cardiac catheterization, cardiac risk score for pediatrics registry, radiation exposure category

## Abstract

**Background:**

The Congenital Cardiac Catheterization Project on Outcomes (C3PO) registry proposed 3-tier radiation exposure categories (REC: I [low], II [medium], and III [high]) consisting of 40 procedure types. This study sought to evaluate the recent trend of radiation exposure in the Catheterization Risk Score for Pediatrics (CRISP) registry organized by the Congenital Cardiovascular Interventional Study Consortium.

**Methods:**

The analysis was conducted on a comprehensive data set from the CRISP registry, covering 13 institutions from January 1, 2016, to December 31, 2020. Radiation dosage in μGym^2^/kg was evaluated by REC, time, and institutions. The study period was divided into the first half (S1: 1/2016-6/2018) and the second half (S2: 7/2018-12/2020). Radiation dosage was compared between S1 and S2. Radiation reduction practices were assessed at participating centers through a questionnaire.

**Results:**

Among 20,524 cases, the majority (n = 18,603, 90.2%) were assigned to C3PO REC procedure types. From S1 (n = 8956) to S2 (n = 9647), median radiation dosage significantly improved in all 3 tiers (*P* < .001): (1) REC I, −18%; (2) REC II, −33%; and (3) REC III, −30%. REC successfully stratified cases by median radiation dosage: (1) REC I, 18.2 μGym^2^/kg (n = 14,234); (2) REC II, 49.8 μGym^2^/kg (n = 3012); and (3) REC III, 67.0 μGym^2^/kg (n = 1357) but showed significant intraclass variability and heterogeneity. REC I exhibited the most variability in radiation dosage. To address these limitations, the procedures were organized into 6 updated REC categories (CRISP REC).

**Conclusions:**

A significant reduction in radiation dosage was observed in the CRISP registry, although a few centers showed a trend of increasing radiation dosage. Despite its limitations, the C3PO REC provides a practical way to stratify cases for reporting dosage. We propose the CRISP REC as a refined alternative to the C3PO REC to improve stratification and decrease variability in radiation exposure across different categories.

## Introduction

The use of x-rays during fluoroscopy and angiography is a fundamental imaging function in the congenital cardiac catheterization laboratory (CCCL). This imaging modality involves radiation exposure to patients and health care providers. Because radiation exposure has a significant stochastic effect, minimizing radiation exposure is an essential goal in the CCCL without compromising diagnosis integrity and procedure safety.^1^, ^2^, ^3^, ^4^, ^5^, ^6^, ^7^ Accordingly, the measurement of radiation exposure dosage is a critical safety metric that needs to be continuously monitored and compared with national benchmark performance over time. However, radiation dosage tracking is challenging due to the highly heterogeneous cardiac catheterization procedures in CCCL.^8^ Previous studies reported the radiation dosage in major procedural categories such as biopsy, diagnostic, interventional catheterizations, and various uniform procedural types.^8^^,^^9^ Recently, the Congenital Cardiac Catheterization Project on Outcomes (C3PO) registry reported 3-tier expected radiation exposure categories (REC: I [low], II [medium], and III [high])—consisting of 40 procedure types based on median dose area product per body weight (DAP/kg, μGym^2^/kg) of <100, 100 to <200, and ≥ 200, respectively.^9^^,^^10^ This study aimed to analyze the recent trend of radiation exposure in the Catheterization Risk Score for Pediatrics (CRISP) registry conducted by the Congenital Cardiovascular Interventional Study Consortium. The study also aimed to assess the usefulness of C3PO REC in categorizing procedures based on the level of radiation exposure in this cohort.

## Materials and methods

The CRISP registry is multicenter prospective observational case registry of congenital cardiac catheterization procedures. The retrospective analysis of radiation exposure dosage was performed during a 5-year study period (January 1, 2016 - December 31, 2020), during which 13 institutions consistently entered the radiation exposure dosage. The radiation exposure dosage metric was DAP/kg (μGym^2^/kg), comprising total cineangiography and fluoroscopy exposure during the procedures. All the cases were included in the analysis, except cases with hybrid procedures (n = 183) and missing weight or radiation dosage. Demographic characteristics, primary case type (biopsy, diagnostic, and interventional), significant adverse event, sheath time (duration between sheath-in and sheath-out), fluoroscopy time, CRISP, and Catheterization Risk Score in Adults (CRISA) score were included for the analysis.

The C3PO registry defined the 40 unique procedure types that were assigned to 91% of cases enrolled in the CRISP registry. These procedure types were grouped into 3-tier C3PO REC categories consisting of I (low: procedure types 1-13), II (medium: 14-29), and III (high: 30-40). Major case types included biopsy (biopsy only and biopsy with coronary angiography), diagnostic (diagnostic catheterization and vasodilator testing), and interventional. Based on an expert consensus, the original 40 unique procedure types were then recategorized into 6 new categories (CRISP REC): (1) biopsy, (2) biopsy + coronary angiography, (3) diagnostic, (4) low, (5) medium, and (6) high interventional categories. Median DAP/kg cut-offs of <30 μGym^2^/kg, 30-60 μGym^2^/kg, and >60 μGym^2^/kg were used to stratify the interventional cases. To assess the change in DAP/kg over time, the study period was split into 2 equal halves: the first half (S1: January 1, 2016 - June 30, 2018) and the second half (S2: July 1, 2018 - December 31, 2020). DAP/kg was evaluated by unique procedure types, C3PO REC, age group, and year of procedure study periods. DAP/kg data had a right-skewed distribution and were expressed as a median with IQR. Data between groups were compared using the χ^2^, Mann-Whitney *U*, or Kruskal-Wallis H tests.

All 13 participating institutions were administered an 11-question survey. Responses to questionnaire items and all abstracted data were entered into REDCap electronic data report forms.

## Results

**Figure 1:**
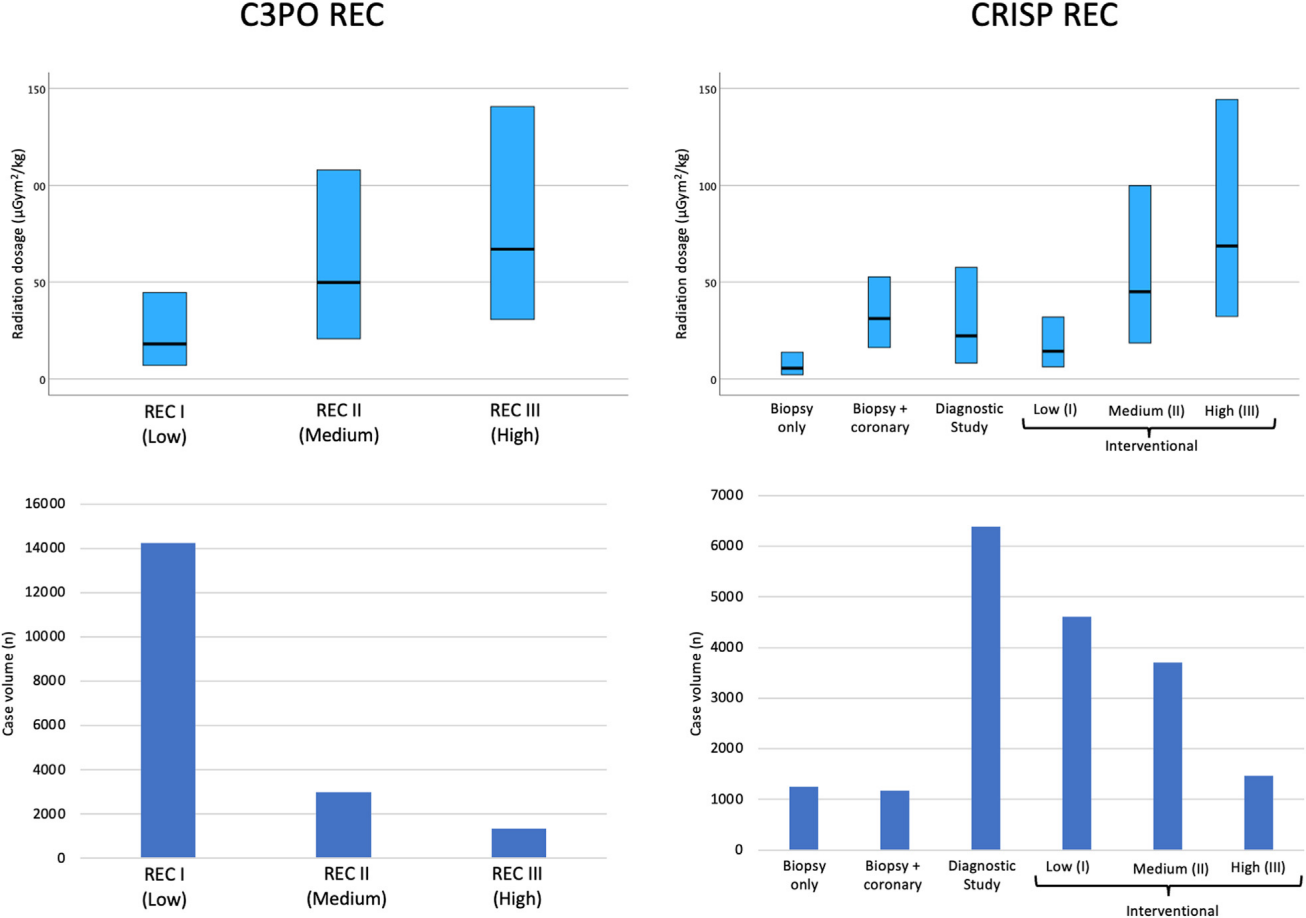
**Comparative box plots of grouped radiation exposure dosage (μGym^2^/kg) using the C3PO radiation exposure categories (C3PO REC) and the CRISP radiation exposure categories (CRISP REC) (top panel).** The bar graph at the bottom panel highlights the case volume in individual categories.

**Figure 2:**
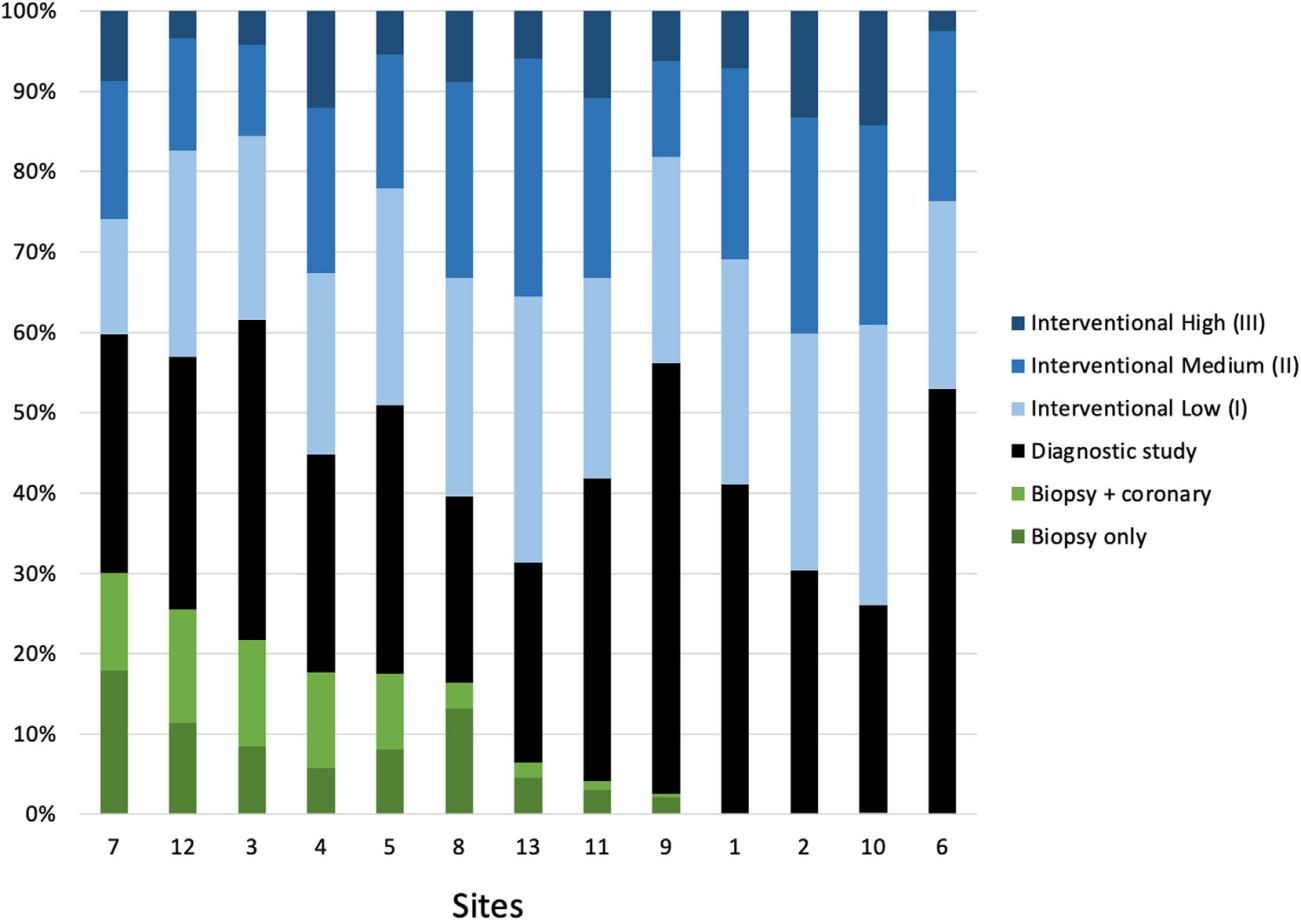
**Distribution of cases in each of the 13 participating institutions based on the CRISP REC**. The graphs are organized in order of decreasing frequency of biopsy volume.

The study included 18,603 cases from 13 institutions (Table 1; Supplemental Table S1). The median overall DAP/kg was 23.4 (9.0 to 59.7) μGym^2^/kg. Most cases involved children, with only 14.7% involving adults. Table 2 shows the number of cases, median DAP/kg, and IQR for 40 unique procedure types. The most common primary case type was interventional (53%), followed by diagnostic (34%) and biopsy (13%; Table 2). Cases were evenly distributed from 2016-2020, with 8956 cases (48%) from S1 and 9647 (52%) from S2. There were slightly more adult subjects in S1 (15.6% vs 13.8%). There were 687 significant adverse events (3.7%). About 3 quarters of cases were categorized as REC I (n = 14,234, 77%), REC II had 3012 cases (16%), and REC III had 1357 cases (7%) using the C3PO categories (Table 2; Central Illustration). When recategorized into CRISP REC categories (Table 3; Figure 1), the diagnostic study was the most common category (34.3%). The most frequent procedure type was diagnostic catheterization (n = 5551); the most common intervention was PDA closure (n = 1806). Certain procedure types had low case volume (n < 20, 0.1%), mostly involving combined interventions. Significantly higher case volumes (n ≥ 20) were found in the following excluded procedure types (9%): systemic vein angioplasty/stent (n = 314), systemic shunt angioplasty/stent (n = 149), systemic artery angioplasty/stent (n = 48), and closure of left superior vena cava (n = 25). A comparison of case mixtures among 13 institutions is shown in Figure 2 and Supplemental Table S1. Seven institutions had low biopsy case volumes, constituting 6% or less, whereas 4 centers reported no biopsy cases at all. In contrast, 6 centers had a substantial volume of biopsies, accounting for more than 15% of total cases. The adult case volume varied between institutions, ranging from 5% to 27%.

**Table 1:** Case mixture and overall radiation exposure.

Characteristic	Value	*P* value
Age group, y		NA
<1	5643 (30.3%)	
1 to <18	10,231 (54.9%)	
≥18	2729 (14.7%)	
Weight, kg	16.3 (7.0-50.2)	NA
Major case type		NA
Biopsy	2429 (13.1%)	
Diagnostic	6380 (34.3%)	
Interventional	9794 (52.6%)	
Radiation exposure category (REC)		NA
REC I	14,234 (76.5%)	
REC II	3012 (16.2%)	
REC III	1357 (7.3%)	
Study period		NA
S1: January 2016 - June 2018	8956 (48.1%)	
S2: July 2018 - December 2020	9647 (51.9%)	
Year		NA
2016	3200 (17.2%)	
2017	3800 (20.4%)	
2018	4042 (21.7%)	
2019	4011 (21.6)	
2020	3550 (19.1%)	
Significant adverse event		NA
Yes	687 (3.7%)	
No	17.916 (96.3%)	
Sheath time, min		<.001
REC I	64 (43-95)	
REC II	117 (83-164)	
REC III	161 (118-215)	
Fluoroscopy time, min		<.001
REC I	12 (7-20)	
REC II	29 (18-44)	
REC III	42 (28-63)	
Radiation dosage, μGym^2^/kg		<.001
REC I	18.1 (7.1-44.7)	
REC II	49.8 (20.9-107.9)	
REC III	67.0 (30.7-140.7)	
Major case type		<.001
Biopsy	15.0 (4.6-35.0)	
Diagnostic	22.4 (8.3-57.6)	
Interventional	27.8 (11.1-71.3)	
Study period		<.001
S1: January 2016 - June 2018	27.2 (9.8-67.5)	
S2: July 2018 - December 2020	21.1 (8.3-52.9)	
Year		<.001
2016	28.8 (10.3-69.8)	
2017	29.1 (11.0-71.7)	
2018	22.3 (8.1-56.6)	
2019	20.5 (7.7-51.1)	
2020	20.4 (8.6-50.9)	
Significant adverse event		<.001
Yes	130.5 (75.8-201.0)	
No	74.0 (48-114.0)	

Values are n (%) or median (IQR).

NA, not applicable.

**Table 2:** Procedure types by C3PO radiation exposure category (C3PO-REC) (N = 18,603).

REC	Procedure type	n	Radiation exposure, μGym^2^/kg
**REC I (low)**			
1	Biopsy	1258	5.6 (2.2-13.9)
2	ASD or PFO closure	1395	11.6 (4.6-25.9)
3	PDA device or coil closure	1806	12.8 (6.1-27.3)
4	Vasodilator testing	829	13.9 (5.4-32.4)
5	Atrial septostomy	270	18.3 (6.4-43.8)
6	Pulmonary valvotomy	674	18.6 (8.2-40.7)
7	Biopsy + CA	1171	31.3 (16.2-52.7)
8	PDA stent placement	149	25.3 (14.9-60.7)
9	Diagnostic catheterization	5551	24.1 (8.9-60.7)
10	Fenestration device closure	46	47.4 (18.2-83.8)
11	Aortic valvotomy	317	25.6 (12.6-54.1)
12	Aorta dilation and or stent	749	33.3 (13.4-75.5)
13	Pulmonary valvotomy + intervention^a^	19	66.7 (29.8-101.6)
**REC II (medium)**			
14	Proximal pulmonary angioplasty or stent	834	47.5 (17.9-99.9)
15	VSD device closure + intervention^a^	79	41.9 (20.0-110.4)
16	RVOT dilation/stent	398	45.4 (19.6-103.4)
17	ASD or PFO closure + intervention^a^	28	37.9 (13.6-124.6)
18	Venous collateral closure	179	53.4 (25.9-110.7)
19	Distal pulmonary angioplasty or stent	252	54.5 (21.1-107.6)
20	Aorta dilation/stent + intervention^a^	100	59.1 (33.0-120.6)
21	Atrial needle transeptal puncture	22	56.9 (20.8-184.0)
22	Atrial Septostomy + intervention^a^	66	44.9 (23.6-107.9)
23	Coil systemic pulmonary collateral	393	43.9 (17.3-100.0)
24	Proximal R and L pulmonary angioplasty	149	60.8 (30.2-132.7)
25	Proximal or distal pulmonary angioplasty or stent + intervention	291	53.2 (24.3-113.6)
26	Atretic valve perforation	14	74.5 (43.9-187.4)
27	Atrial septum stent placement	53	56.8 (24.6-115.0)
28	Fenestration device closure + intervention^a^	28	91.4 (46.5-146.9)
29	RVOT dilation or stent + proximal pulmonary angioplasty or stent	126	61.5 (30.2-132.3)
**REC III (high)**			
30	Mitral valvotomy + intervention^a^	13	48.4 (25.1-120.1)
31	TPV replacement	542	68.6 (31.8-162.6)
32	≥ 2 vessel proximal or distal angioplasty or stent	155	68.6 (29.8-123.9)
33	Coil systemic pulmonary collateral + intervention^a^	168	41.2 (21.0-108.9)
34	Aortic valvotomy + intervention^a^	16	46.3 (18.8-121.9)
35	RVOT dilation/stent and ≥ 2 vessel proximal or distal pulmonary angioplasty or stent	23	46.0 (27.5-125.0)
36	TPV replacement and PA Intervention^a^	64	89.1 (56.6-182.0)
37	≥ 2 vessel proximal or distal pulmonary angioplasty or stent + intervention	53	84.1 (25.4-227.2)
38	Pulmonary vein dilation or stent	263	68.9 (34.6-130.9)
39	TPV replacement + intervention^a^	9	112.9 (46.7-152.5)
40	Pulmonary vein dilation or stent + intervention^a^	51	80.0 (40.9-148.1)

Values are median (IQR) unless otherwise stated.

ASD, atrial septal defect; CA, coronary angiography; L, left; PA, pulmonary artery; PDA, patent ductus arteriosus; PFO, patent foramen ovale; PS, pulmonary stenosis; R, right; REC, radiation exposure category; RVOT, right ventricular outflow tract; TPV, transcatheter pulmonary valve placement; VSD, ventricular septal defect.

a Intervention defined as additional angioplasty and/or stent placement, valvuloplasty, transeptal needle puncture, or coiling of systemic or venous collateral vessel.

**Central Illustration:**
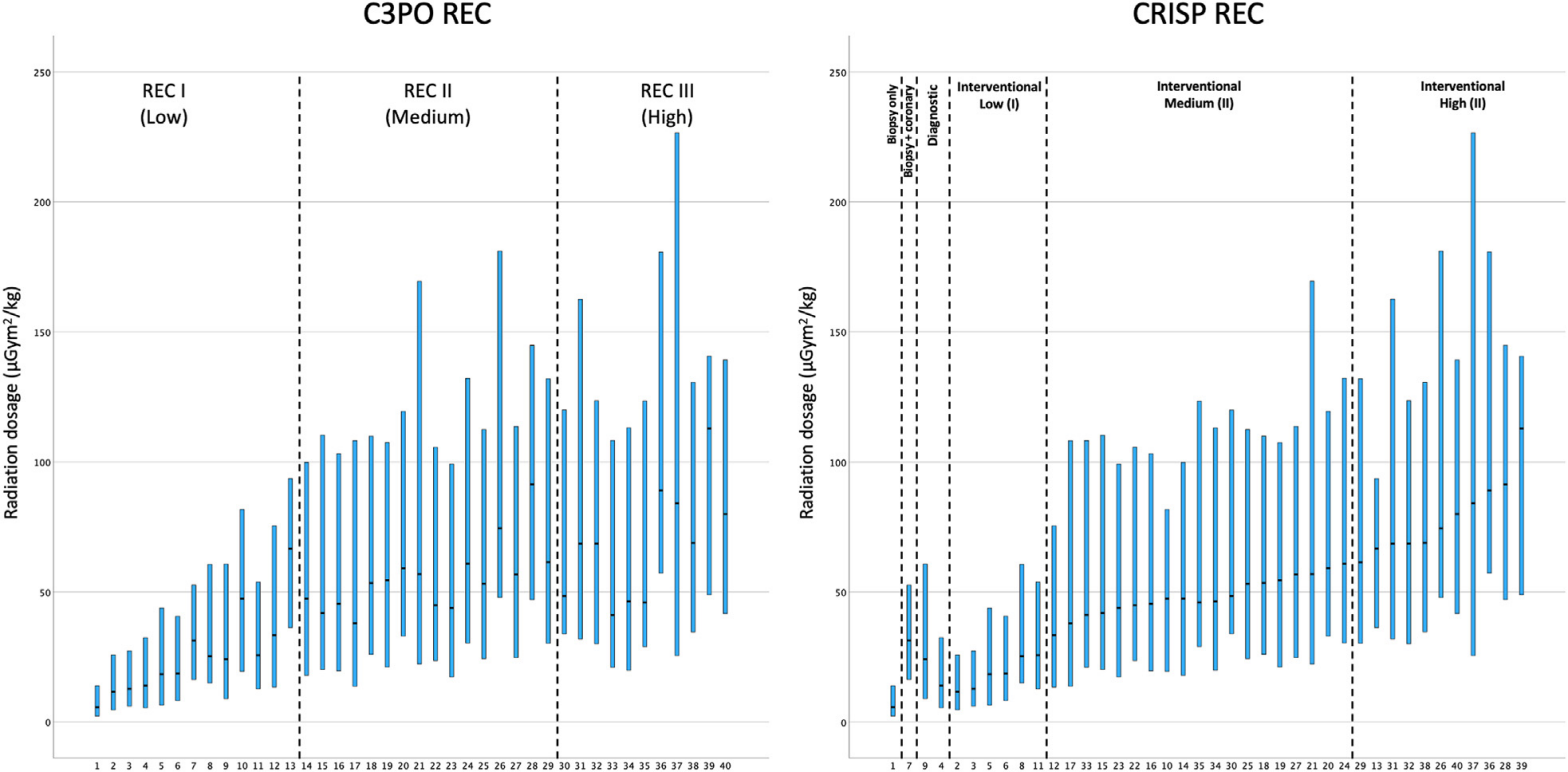
Box plot of radiation exposure dosage (μGym^2^/kg) organized by individual procedure types using the C3PO radiation exposure category (C3PO REC) and the CRISP radiation exposure category (CRISP REC). The black dot indicates the median value, and the box indicates the interquartile range.

**Table 3:** Procedure types by CRISP radiation exposure category (CRISP REC) (N = 18,603).

Procedure types		n	Radiation exposure, μGym^2^/kg
**Biopsy**			
1	Biopsy	1258	5.6 (2.2-13.9)
**Biopsy + Coronary study**			
7	Biopsy + CA	1171	31.3 (16.2-52.7)
**Diagnostic study**		6380	22.4 (8.3-57.6)
9	Diagnostic catheterization	5551	24.1 (8.9-60.7)
4	Vasodilator testing	829	13.9 (5.4-32.4)
**Interventional low (I)(<30 μGym^2^/kg)**		4611	14.4 (6.3-32.0)
2	ASD or PFO closure	1395	11.6 (4.6-25.9)
3	PDA device or coil closure	1806	12.8 (6.1-27.3)
5	Atrial septostomy	270	18.3 (6.4-43.8)
6	Pulmonary valvotomy	674	18.6 (8.2-40.7)
8	PDA stent placement	149	25.3 (14.9-60.7)
11	Aortic valvotomy	317	25.6 (12.6-54.1)
**Interventional medium (II) (30-60μGym^2^/kg)**		3710	45.0 (18.6-99.9)
12	Aorta dilation and or stent	749	33.3 (13.4-75.5)
17	ASD or PFO closure + intervention	28	37.9 (13.6-124.6)
33	Coil systemic pulmonary collateral + intervention	168	41.2 (21.0-108.9)
15	VSD device closure + intervention	79	41.9 (20.0-110.4)
23	Coil systemic pulmonary collateral	393	43.9 (17.3-100.0)
22	Atrial Septostomy + intervention	66	44.9 (23.6-107.9)
16	RVOT dilation/stent	398	45.4 (19.6-103.4)
10	Fenestration device closure	46	47.4 (18.2-83.8)
14	Proximal pulmonary angioplasty or stent	834	47.5 (17.9-99.9)
35	RVOT dilation/stent and ≥ 2 vessel proximal or distal pulmonary angioplasty or stent	23	46.0 (27.5-125.0)
34	Aortic valvotomy + intervention	16	46.3 (18.8-121.9)
30	Mitral valvotomy + intervention	13	48.4 (25.1-120.1)
25	Proximal or distal pulmonary angioplasty or stent + intervention	291	53.2 (24.3-113.6)
18	Venous collateral closure	179	53.4 (25.9-110.7)
19	Distal pulmonary angioplasty or stent	252	54.5 (21.1-107.6)
27	Atrial septum stent placement	53	56.8 (24.6-115.0)
21	Atrial needle transeptal puncture	22	56.9 (20.8-184.0)
20	Aorta dilation/stent + intervention	100	59.1 (33.0-120.6)
**Interventionalhigh (III) (>60 μGym^2^/kg)**		1473	68.7 (32.3-144.6)
24	Proximal R and L pulmonary angioplasty	149	60.8 (30.2-132.7)
29	RVOT dilation or stent + proximal pulmonary angioplasty or stent	126	61.5 (30.2-132.3)
13	Pulmonary valvotomy + intervention	19	66.7 (29.8-101.6)
31	TPV replacement	542	68.6 (31.8-162.6)
32	≥ 2 vessel proximal or distal angioplasty or stent	155	68.6 (29.8-123.9)
38	Pulmonary vein dilation or stent	263	68.9 (34.6-130.9)
26	Atretic valve perforation	14	74.5 (43.9-187.4)
40	Pulmonary vein dilation or stent + intervention	51	80.0 (40.9-148.1)
37	≥ 2 vessel proximal or distal pulmonary angioplasty or stent + intervention	53	84.1 (25.4-227.2)
36	TPV replacement and PA Intervention	64	89.1 (56.6-182.0)
28	Fenestration device closure + intervention	28	91.4 (46.5-146.9)
39	TPV replacement + intervention	9	112.9 (46.7-152.5)

Values are median (IQR) unless otherwise stated.

Procedure numbers are unchanged from Table 2 and are arranged in increasing order of median radiation dose in each category. + Intervention defined as additional angioplasty and/or stent placement, valvuloplasty, transeptal needle puncture, or coiling of systemic or venous collateral vessel.

ASD, atrial septal defect; CA, coronary angiography; L, left; PA, pulmonary artery; PDA, patent ductus arteriosus; PFO, patent foramen ovale; PS, pulmonary stenosis; R, right; RVOT, right ventricular outflow tract; TPV, transcatheter pulmonary valve placement; VSD, ventricular septal defect.

Higher C3PO REC was associated with significantly longer sheath and fluoroscopy times (*P* < .001). Dose area product was highest in REC III (median 67.0 μGym^2^/kg), compared with REC II (49.8 μGym^2^/kg) and REC I (18.1 μGym^2^/kg). Interventional cases had higher DAP/kg than diagnostic and biopsy cases. From 2016 to 2020, the median DAP/kg decreased gradually from 29 to 20 μGym^2^/kg. S2 had significantly a lower median DAP/kg than S1 (21 vs 27 μGym^2^/kg, *P* < .001). Overall, cases with significant adverse events had much higher DAP/kg. The box plot in the Central Illustration depicts the radiation exposure dosage for 40 unique procedure types categorized by both C3PO and CRISP REC. In the C3PO system, the 13 REC I procedure types had lower median DAP/kg with a much narrower IQR. In contrast, the 16 REC II and 11 REC III procedure types significantly overlap in median DAP/kg with wider IQR. The CRISP REC system provided slightly improved stratification of interventional procedure types by reorganizing them based on median DAP/kg. However, substantial overlap between interventional medium (II) and High (III) procedure types remained. A key distinction in the CRISP REC is the separation of biopsy (with or without coronary study) and diagnostic procedures from the low (I) interventional category, enhancing procedural classification.

**Figure 3:**
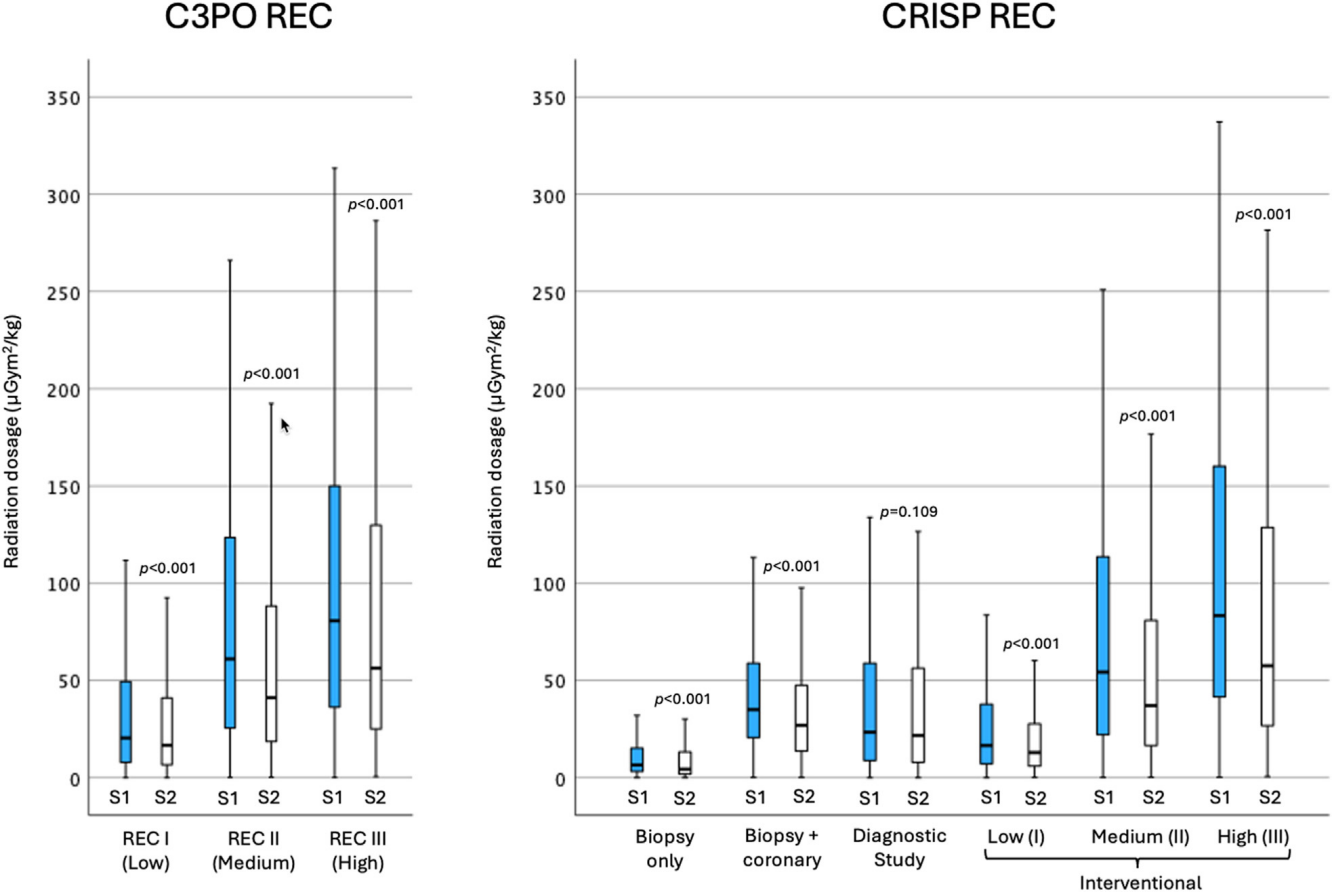
**Box and whisker plot to compare radiation dosage (μGym^2^/kg) between study period 1 (S1: January 2016 - June 2018) and period 2 (S2: July 2018 - December 2020), stratified by the C3PO radiation exposure categories (C3PO REC) and the CRISP radiation exposure categories (CRISP REC)**. Data were compared using the Mann-Whitney *U* test.

**Figure 4:**
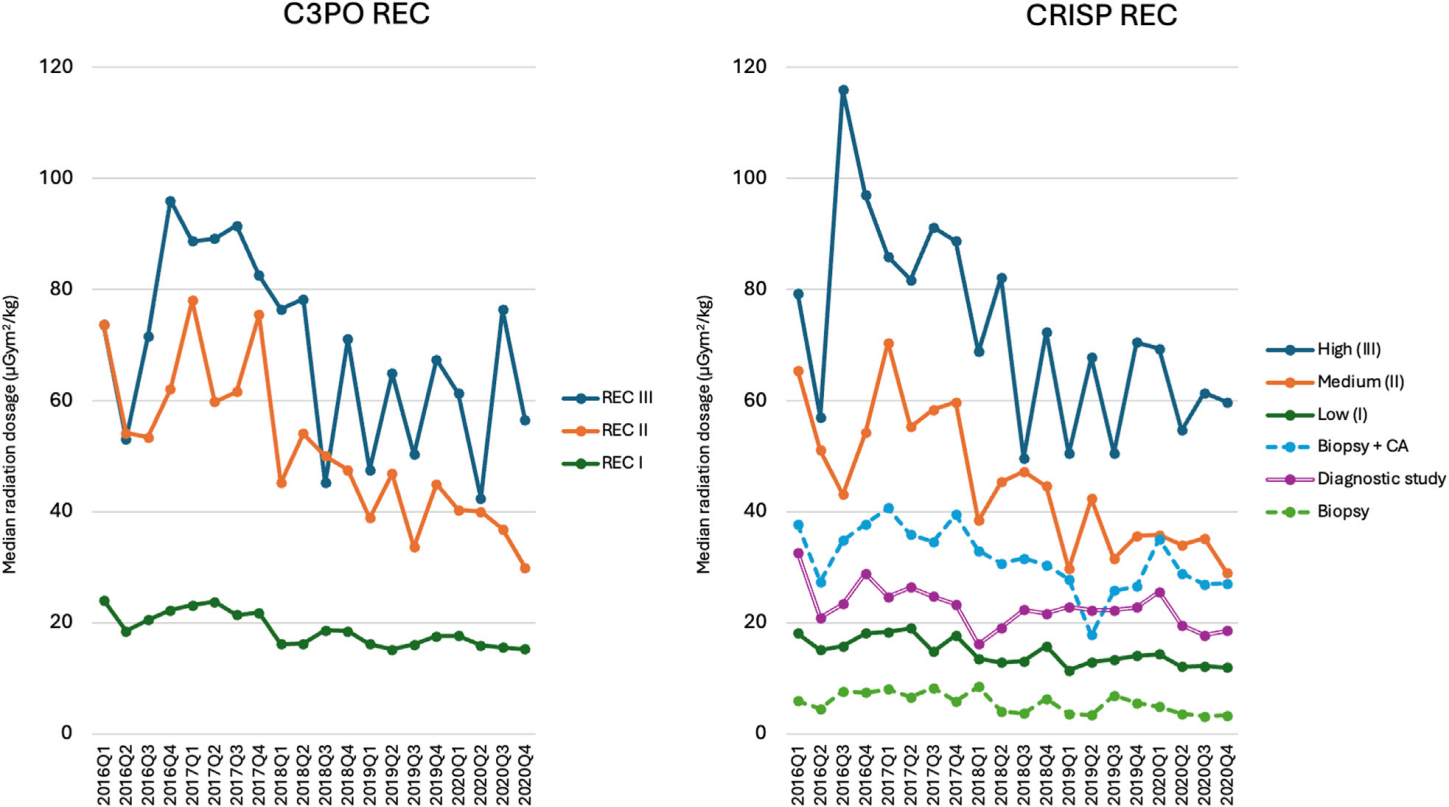
**Quarterly trend of radiation dosage from 2016-2020, stratified by C3PO radiation exposure categories (C3PO REC) and the CRISP radiation exposure categories (CRISP REC).**

A significant reduction in radiation exposure dosage was observed over the study period (Table 4). In each C3PO REC, adult cases had twice as high DAP/kg as cases involving infants/children (Supplemental Table S2). From S1 to S2, the median radiation dosage significantly decreased (*P* < .001) by 18% in C3PO REC I, 33% in C3PO REC II, and 30% in C3PO REC III (Figure 3). This trend of reduced radiation doses was also observed within the CRISP REC classification over the 2 study periods, except for the diagnostic study group. Upon reclassification, the CRISP REC categories—biopsy only, biopsy + coronary, diagnostic study, and low interventional group—showed less variability compared with the C3PO REC low (I) category (Figure 3). The trend of radiation dosage reduction was similarly observed yearly from 2016-2020 in each C3PO and CRISP REC (Figure 4). The change in radiation exposure dosage was observed in most institutions (Table 4). Comparisons of patient and procedural characteristics affecting the reduction in radiation exposure showed no clinically meaningful difference between the 2 study periods (Supplemental Table S3).

**Table 4:** Reduction of radiation dosage from study period 1 (January 2016 - June 2018) to period 2 (July 2018 - December 2020), stratified by REC per institution.

Site	REC	Study period 1 (S1) January 2016 - June 2018		Study period 2 (S2) July 2018 - December 2020		% change from S1 to S2
		n	Radiation exposure, μGym^2^/kg	n	Radiation exposure, μGym^2^/kg	
1	I	570	21.6 (10.8-51.5)	528	21.2 (11.2-45.9)	−1.9%
	II	132	53.7 (26.8-82.5)	158	44.1 (23.9-73.3)	−17.9%
	III	52	56.5 (33.8-138.3)	47	66.7 (44.8-157.9)	+15.3%
2	I	337	67.1 (30.6-130.3)	358	44.5 (22.0-77.0)	−33.7%
	II	128	135.1 (87.3-244.2)	125	96.1 (46.6-144.5)	−28.9%
	III	49	206.9 (130.4-356.2)	59	143.5 (102.3-226.5)	−30.6%
3	I	392	43.0 (23.9-72.7)	469	32.7 (17.5-56.3)	−24.0%
	II	39	109.8 (67.4-166.3)	42	74.9 (54.1-134.7)	−31.8%
	III	23	174.0 (89.1-226.0)	16	129.3 (105.9-223.2)	−25.7%
4	I	959	10.1 (4.3-20.2)	1000	7.6 (3.3-17.1)	−24.8%
	II	206	24.4 (14.9-40.7)	220	19.0 (11.4-35.5)	−22.1%
	III	152	41.6 (25.7-75.8)	196	29.7 (16.7-56.1)	−28.6%
5	I	793	31.5 (15.2-59.5)	697	29.3 (16.2-54.0)	−7.0%
	II	147	97.8 (56.1-145.7)	106	80.6 (55.9-124.2)	−17.6%
	III	37	141.4 (86.2-244.4)	50	108.1 (66.8-143.1)	−23.6%
6	I	520	38.3 (12.8-90.0)	505	70.2 (34.5-130.7)	+83.3%
	II	142	109.0 (60.9-182.0)	68	156.1 (74.2-240.9)	+43.2%
	III	19	166.4 (4.5-199.3)	5	323.8 (149.0-830.8)	+94.6%
7	I	970	21.1 (8.3-47.9)	881	8.8 (3.5-18.4)	−58.3%
	II	175	61.0 (345-110.1)	198	22.8 (13.8-39.9)	−62.6%
	III	73	122.8 (63.3-253.5)	101	37.5 (21.2-65.3)	−69.5%
8	I	446	27.9 (11.2-68.8)	553	20.8 (8.6-42.3)	−25.4%
	II	140	91.3 (54.3-157.6)	160	67.0 (40.0-124.7)	−26.6%
	III	34	117.6 (64.0-235.0)	44	149.1 (95.6-262.1)	+26.8%
9	I	562	9.5 (5.4-16.5)	636	10.2 (5.3-21.3)	+7.4%
	II	69	21.8 (13.1-47.2)	68	27.5 (18.3-51.0)	+26.2%
	III	35	61.7 (39.5-90.8)	55	60.2 (32.8-97.8)	−2.4%
10	I	115	15.1 (7.6-33.2)	208	27.9 (11.6-71.7)	+84.8%
	II	31	116.4 (38.3-176.1)	58	141.6 (61.1-240.4)	+21.7%
	III	19	101.0 (58.5-150.3)	46	187. 0 (119.8-370.5)	+85.1%
11	I	342	8.3 (3.6-14.8)	512	5.0 (2.3-9.6)	−39.8%
	II	74	18.0 (11.1-33.3)	130	16.4 (10.8-32.2)	−8.9%
	III	51	35.4 (22.2-69.9)	74	29.1 (10.7-48.5)	−17.8%
12	I	432	34.4 (3.4-63.9)	573	22.5 (6.6-56.5)	−34.6%
	II	58	119.3 (68.7-188.1)	63	91.0 (51.4-133.1)	−23.7%
	III	14	118.0 (74.2-352.9)	32	89.1 (56.7-192.3)	−24.5%
13	I	392	8.8 (2.9-31.2)	484	11.5 (5.3-23.9)	+30.7%
	II	175	29.8 (12.4-70.3)	100	15.4 (8.6-26.9)	−48.3%
	III	52	89.4 (46.7-122.3)	26	17.3 (10.6-43.7)	−80.6%

Values are median (IQR) unless otherwise noted.

Factors affecting DAP/kg were examined in each C3PO REC. The impact of adult cases was significant. In each REC, the median DAP/kg in adult cases was nearly twice as high as that in children. In contrast, median DAP/kg was comparable between infants (<1-year-old) and children (<18-year-old). In cases with significant adverse events, DAP/kg was higher in each REC. The higher CRISP/CRISA category had higher DAP/kg in each REC (Table 5). Median DAP/kg in all 3 radiation exposure categories improved in adults over the 2 study periods (−20.2%, −25.7%, and −36.9% in REC I, II, and III, respectively, Supplemental Table S3). These improvements were similar to those observed in children (−17.4%, −32.1%, and −24.4% in REC I, II, and III, respectively). The most significant unique procedure type case volume was diagnostic catheterization (n = 5551). In this large group of cases, 976 (17.6%) were adults. Higher radiation dosage in adult cases was also observed in other large-volume unique procedure types such as atrial septal defect/patent foramen ovale closure, proximal pulmonary artery angioplasty or stent, and transcatheter pulmonary valve replacement (Supplemental Table S4). The sheath and fluoroscopy time did not change significantly over the study period in our data set (Supplemental Table S5).

**Table 5:** Impact of CRISP/CRISA category on radiation dosage, SAE, sheath time, fluoroscopy time, stratified by CRISP radiation exposure category (REC).

Clinical factors	Cases (n)	Radiation exposure, μGym^2^/kg	SAE rate	Sheath time, min	Fluoroscopy time, min
**Biopsy**					
CRISP category (<18 y)					
1	620	5.6 (2.1-13.6)	0.6%	27 (20-36)	5 (3-8)
2	436	4.7 (2.2-13.3)	1.6%	33 (26-47)	7 (5 -11)
3	46	9.3 (3.5-22.1)	4.3%	36 (26-75)	9 (7-15)
4	3	12.6 (NA)	0%	29 (NA)	8 (NA)
5	0	NA	NA	NA	NA
CRISA category (≥18 y)					
1	24	7.1 (5.8-20.4)	0%	31 (23-48)	4 (3-9)
2	126	6.0 (2.5-15.8)	2.4%	28 (22- 42)	6 (3-9)
3	2	13.8 (NA)	0%	89 (NA)	19
4	1	NA	0%	NA	
**Biopsy + CA**					
CRISP category (<18 y)					
1	344	23.8 (12.5-45.2)	1.7%	55 (44-67)	11 (8-16)
2	544	31.6 (18.2-49.3)	1.8%	66 (55-82)	15 (11-19)
3	21	43.9 (20.5-81.0)	9.5%	74 (66-88)	16 (11-20)
4	1	NA	0%	NA	NA
5	0	NA	NA	NA	NA
CRISA category (≥18 y)					
1	22	48.4 (25.2-65.4)	0%	58 (51-74)	13
2	236	37.9 (19.2-66.3)	1.3%	60 (49-75)	13
3	1	NA	0%	NA	NA
4	2	NA	0%	NA	NA
**Diagnostic study**					
CRISP category (<18 y)					
1	2070	20.2 (7.5-50.1)	1.2%	64 (43-92)	11 (7-18)
2	1799	18.5 (7.3-44.1)	2.4%	77 (54-107)	15 (9-24)
3	1172	21.6 (8.8-52.5)	4.9%	78 (55-109)	15 (9-25)
4	257	24.5 (11.6-50.1)	11.7%	88 (57-124)	19 (11-28)
5	6	89.7 (14.4-338.5)	33.3%	183 (106-235)	55 (27-107)
CRISA category (≥18 y)					
1	344	34.0 (10.1-90.0)	0.9%	65 (44-100)	12 (7-21)
2	701	50.2 (16.1-109.0)	2.7%	73 (52-108)	14 (8-22)
3	29	54.6 (8.1-114.3)	3.4%	121 (65-153)	18 (8-31)
4	2	NA	0%	NA	NA
**Interventional low (I)**					
CRISP category (<18 y)					
1	56	11.1 (5.3-22.2)	0%	62 (40-111)	12 (7-22)
2	2147	10.7 (4.7-22.2)	2.5%	57 (42-80)	11 (7-17)
3	1303	15.7 (7.4-37.5)	4.5%	59 (37-90)	11 (7-19)
4	602	21.7 (9.7-44.7)	8.3%	68 (45-105)	15 (9-26)
5	50	23.6 (10.2-59.6)	18.9%	101 (59-135)	19 (13-33)
CRISA category (≥18 y)					
1	6	88.1 (31.6-135.3)	0%	59 (38-83)	14 (11-22)
2	363	27.0 (12.1-56.8)	5.0%	66 (46-105)	12 (8-20)
3	73	32.0 (13.9-82.4)	1.4%	73 (49-106)	15 (10-24)
4	10	63.6 (42.1-296.4)	0%	72 (60-110)	23 (15-24)
**Interventional medium (II)**					
CRISP category (<18 y)					
1	39	29. 7 (14.4-97.5)	0%	120 (92-215)	30 (15-43)
2	696	45.9 (17.6-95.8)	2.3%	111 (79-155)	26 (16-41)
3	1882	38.6 (16.9-89.7)	4.1%	110 (79-155)	24 (15-39)
4	647	44.8 (20.0-91.3)	6.8%	107 (73-153)	27 (16-44)
5	51	46.0 (21.1-101.8)	13.7%	110 (69-172)	26 (16-45)
CRISA category (≥18 y)					
1	2	NA	0%	NA	NA
2	318	84.3 (37.1-158.0)	4.4%	120 (85-172)	25 (16-38)
3	67	129.7 (42.4-216.7)	7.5%	148 (108-191)	33 (18-46)
4	8	65.4 (41.6-111.5)	12.5%	134 (102-187)	32 (21-50)
**Interventional high (III)**					
CRISP category (<18 y)					
1	0	NA	NA	NA	NA
2	42	52.2 (21.7-89.6)	9.5%	130 (79-164)	30 (19-39)
3	711	61.0 (28.5-124.6)	4.8%	149 (108-201)	38 (26-57)
4	300	71.0 (34.9-140)	14.7%	157 (119-210)	51 (33-109)
5	28	80.9 (47.6-146.0)	10.7%	166 (99-223)	45 (31-83)
CRISA category (≥18 y)					
1	0	NA	NA	NA	NA
2	315	85.7 (41.8-221.5)	6.3%	162 (126-227)	39 (25-56)
3	69	110.2 (45.0-186.4)	15.9%	175 (122-241)	39 (27-58)
4	8	66.9 (26.2-99.5)	0%	120 (101-212)	33 (21-44)

Values are median (IQR) unless otherwise noted. CRISA, Catheterization Risk Score in Adults; CRISP, Catheterization Risk Score for Pediatrics.

All 13 institutions’ questionnaire responses were received (Supplemental Table S6). Only 3 institutions had an active radiation reduction QI initiative, and 2 institutions decreased their default frame rate during the study period. Six institutions used a fluoroscopy frame rate of less than 6 pulses per second, and 11 used a default cine frame rate of 15 fps. Two institutions made changes to baseline default settings during the study period. Six (46%) institutions underwent a replacement or upgrade of their catheterization equipment during the study period. Eleven (85%) institutions have trainees in their laboratories. Eight institutions use a single plane even when a biplane is available for specific procedures. Most institutions (9, 69%) have institutional policies for patient follow-up after a predetermined cutoff for high radiation exposure.

## Discussion

Radiation exposure is an integral part of the current CCCL, but it carries risks, including deterministic and stochastic effects.^11^ There is increasing evidence linking radiation exposure to cancer in both children and interventional cardiologists.^3^^,^^12^, ^13^, ^14^ With a better understanding of the harmful effects of radiation, interventional cardiologists have made concerted efforts to reduce radiation exposure in the CCCL.^1^^,^^5^^,^^15^, ^16^, ^17^, ^18^ Our data showed a significant reduction in radiation exposure dosage in the CCCL during a recent study period (2016-2020) based on a large cohort of 5-year data from 13 institutions.

The C3PO registry initially proposed the REC to stratify common procedure types into categories with similar radiation exposure levels. They proposed 40 unique procedure types grouped into a 3-tier REC based on DAP/kg of <100, 100 to <200, and ≥ 200, respectively.^9^ The rationale behind the C3PO REC was to establish a fair risk adjustment methodology that enables stratified assessment of radiation risk and exposure across institutions for outcome evaluation. However, a fundamental limitation of this approach is the significant variation in radiation dosage across different procedure types within each C3PO REC. For instance, in C3PO REC I (low), which includes 13 procedure types, the median DAP/kg ranged widely from 5 to 99.^9^ In C3PO REC II (medium), encompassing 16 procedures, the median DAP/kg spanned from 108 to 190. Similarly, C3PO REC III (high) included 11 procedures with a median DAP/kg ranging from 201 to 381. Another limitation is that this approach does not account for variations in case type distribution between institutions. Within each REC, centers have different procedural mixes, and consolidating them into a single category may obscure the impact of case composition, leading to an inaccurate representation of radiation exposure across institutions. A particularly striking example is the proportion of biopsies in REC I, which varies significantly across institutions. Biopsies, being the lowest radiation procedure, could unfavorably skew the centers not perform biopsies. The same is true of diagnostic cases.

Although 91% of the cases in the CRISP registry could be categorized under the C3PO REC procedure types, there is still significant variability in the radiation doses within these categories. This variability is most pronounced in the C3PO REC I category, which accounts for 77% of the cases and shows a radiation dose variation of up to 10 times. There is also significant overlap in the radiation dosage exposure between REC categories II and III. To address these limitations, we reclassified the 40 unique procedure types into 6 refined CRISP REC, incorporating both procedural characteristics and radiation doses, as shown in Table 3, Central Illustration, and Figure 1. This refined categorization offers several advantages. Separating biopsy cases from the interventional low category improves stratification and allows for a fairer comparison between heart transplant and non-heart transplant centers. Diagnostic studies were the most frequently performed procedure type and demonstrated significant heterogeneity, as indicated by a much wider IQR than the interventional low category. The new CRISP REC classification has minimized variability in radiation dosage across different categories. By separating diagnostic studies from the interventional low category, we have effectively reduced the variability in radiation dose exposure within the interventional low category, which still maintains the largest case volume among the interventional categories (low, medium, and high). We believe that grouping procedures into simplified and recognizable CRISP REC enhances the C3PO REC classification, enabling more accurate comparisons across institutions with varying frequencies of biopsy and diagnostic procedures. It is important to note that among excluded cases (9%), we observed procedure types with significant case volume (n ≥ 20), such as systemic vein angioplasty/stent (n = 314), systemic shunt angioplasty/stent (n = 149), systemic artery angioplasty/stent (n = 48), and closure of left superior vena cava (n = 25). Analyzing whether opportunities exist to include the excluded procedures, characterizing radiation exposure by other procedural characteristics, and reclassifying procedure types further would be the focus of future studies and newer versions of the CRISP registry, potentially enhancing the usefulness of REC even more.

Various factors, including procedural techniques and patient characteristics, can influence the amount of radiation exposure. CRISP and CRISA scores have been developed and validated to predict the risk of significant adverse events in individual cases.^19^^,^^20^ The risk scores can be used to categorize the complexity of patient characteristics and procedural difficulty. We studied how the CRISP/CRISA category affected radiation exposure in each REC (Table 5). Our findings show that higher CRISP/CRISA risk categories were associated with a higher median DAP/kg and a higher rate of significant adverse events. Although adjusting for patient complexity might provide a more accurate model for categorizing radiation exposure, the complex methodology makes its clinical application challenging. Despite these limitations, C3PO REC remains a valuable tool due to its simplicity.

In our data set, we observed a substantial impact of adult cases on radiation dosage. The proportion of adults in this cohort (14.7%) is slightly higher than reported in the recent C3PO registry (11%); reassuringly, the magnitude of reduction in radiation dosage was similar in adults and children over the study period.^10^ Adult cases exhibited twice the DAP/kg compared with infant/children’s cases in each C3PO REC. This difference could be attributed to various factors, such as the patient’s weight, body habitus, and anatomical changes in the chest with age, which may necessitate higher radiation doses in certain imaging views and procedure types.^21^ We noted higher radiation dosage for large-volume unique procedure types in adult cases, including atrial septal defect/patent foramen ovale closure, proximal pulmonary artery or stent, and transcatheter pulmonary valve replacement. Quinn et al^9^ also investigated the influence of age on radiation dosage. They found that the median DAP/kg was highest at 65 μGym^2^/kg in adults (>19 years), compared with 28 μGym^2^/kg in children for low radiation procedures. However, they did not consider the age factor in their study's REC methodology. Given that adults with congenital heart disease constitute a growing population, this group requires special attention.^12^^,^^13^^,^^22^ Our study highlighted higher radiation exposure associated with age, emphasizing the need for future research to develop an enhanced methodology that accounts for the effect of age on radiation dosage.

There was no significant difference in DAP/kg between infants and children in our cohort when classified by C3PO REC. Although these findings may be reassuring from a radiation safety perspective, this is not what was observed by Quinn et al.^9^ The distribution of patients in their study (1-18 year category at 58% with proportionally fewer patients in the age groups of < 1 year and ≥ 19 years at 24% and 10%, respectively) could have impacted their findings when comparing more common low REC cases. It is imperative to continue building on this framework to understand if younger age and low REC procedure types impose higher radiation exposure at a certain age.

Radiation exposure dosage continues to decline in the CCCL. In a recent report from the C3PO registry, the median DAP/kg decreased by 23% to 37% in REC I-III from 2015 to 2017.^10^ The CRISP registry data set also showed a significant reduction in median DAP/kg by 18% to 33% from 2016-2020 and by REC. The median DAP/kg in the CRISP cohort (18.1, 49.8, and 67 μGym^2^/kg in REC I, II, III respectively) is lower than the median DAP/kg reported by the original C3PO cohort (39, 131, and 231 μGym^2^/kg in REC I, II, and III respectively),^9^ and the more recent C3PO report (27, 106, and 197 μGym^2^/kg in REC I, II, and III, respectively).^10^ The updated CRISP REC applies lower radiation dose cutoffs of <30, 30 to 60, and >60 μGym^2^/kg compared with C3PO REC, reflecting contemporary improvements in radiation reduction strategies and setting a more relevant benchmark for ongoing quality improvement efforts. This framework acknowledges that radiation exposure is a moving target, likely to decrease further with advancements in medical technology and procedural techniques.

The reduction in documented radiation exposure through this registry is a positive development. We attempted to determine the main reasons for this change. Patient and procedural factors such as age, weight, CRISP/CRISA category, REC category, case type, sheath time, and fluoroscopy times showed no significant differences between the 2 study periods. However, the results of the questionnaire revealed interesting observations. During the study period, 6 of the 13 participating centers upgraded their catheterization laboratory angiographic systems, with 1 upgrade occurring at the very beginning and another at the very end. Although no clear inflection points are linked to the upgrades, these advancements may have played a significant role in reducing radiation. The latest generation of angiographic systems employs improved hardware and software that lower radiation usage while enhancing image quality.^11^ Best practice guidelines for reducing radiation exposure emphasize the “ALARA” guiding principle (as low as reasonably achievable). These include techniques such as minimizing fluoroscopy time, using default fluoroscopy and cine frame rates, optimizing collimation and magnification, and employing the best techniques for distance, angulation, and table position.^5^^,^^11^ Most centers reported using default fluoroscopy frame rates of less than 8 pulses per second and default cine frame rates of less than 15 frames per second, which have been shown to reduce radiation exposure without compromising safety.^1^^,^^15^ During the study period, 3 centers had an active quality improvement initiative for radiation reduction, and the operators’ efforts and procedural efficiency likely contributed to the overall trend. The CRISP registry provided radiation dosage monitoring function on the registry website and in the periodic reports using REC as a benchmark, allowing participants to track their local performance, share learning, and enhance engagement in quality improvement efforts including providing maintenance of certification part IV credits through the American Board of Pediatrics for radiation reduction. Although there was no CRISP registry-specific collaborative initiative to reduce radiation exposure dosage during the study period, the radiation dosage monitoring function and maintenance of certification credits provided valuable support for quality improvement efforts.

A concerted effort is needed to improve the quality of care and reduce radiation exposure in the catheterization laboratory. Interventional cardiology societies like the Society of Cardiac Angiography and Interventions have led these efforts with consensus statements and by providing toolkits and tips.^16^^,^^23^^,^^24^ By addressing current challenges, implementing systematic risk categorization, and fostering collaboration, health care professionals can work toward enhancing patient safety and finding the right balance between the diagnostic benefits and potential risks associated with ionizing radiation in congenital heart disease interventions. Ongoing collaborative efforts and technological innovations will be crucial in shaping the future landscape of age-tailored radiation dose optimization in pediatric cardiology. Additionally, these findings will guide interventional cardiologists aiming to achieve the lowest reasonably possible radiation doses.

### Limitations

This study had limitations due to the availability of a limited number of centers with verifiable data collection and its retrospective nature. Since participation in the registry was voluntary, there were no random site visits to audit procedures for accuracy. The generalizability of the findings is affected by the diversity of practices and quality improvement initiatives in this multicenter study. Despite the information from questionnaires, we could not analyze the impact of practice patterns, quality improvement initiatives, and equipment changes on radiation reduction due to their heterogeneity and variable timeline. The impact of newer technologies, such as 3 dimensional rotational angiography, digital subtraction angiography, computed tomography overlay, and the increasing complexity of interventions being performed in the CCCL, is not addressed in this retrospective study design. Although the proposed CRISP REC enhances intraclass homogeneity and facilitates cross-institutional comparisons, it may complicate interpretation for smaller centers by decreasing the number of procedures in each category.

## Conclusion

The institutions involved in the CRISP registry have made significant strides in radiation dosage in recent years. Although the REC by C3PO provided a practical method for stratifying cases to report radiation dosage, it has some limitations. To address these, we propose the CRISP REC as a more refined alternative to the C3PO REC, which reduces variability in radiation dosage across categories. The CRISP registry plans to implement a registry-based quality improvement initiative as part of a concerted effort to reduce radiation exposure further. Our proposed CRISP REC could serve as a valuable framework for evaluating site performance and monitoring progress within the registry.
