# Supplementary material for: Trends in Radiation Exposure With the Refinement of Radiation Exposure Categories in Congenital Cardiac Catheterization: Insights From the CRISP Registry
Source: J Soc Cardiovasc Angiogr Interv. 2025 Jul 23;4(8):103727. doi: 10.1016/j.jscai.2025.103727 (PMC12462090; doi:10.1016/j.jscai.2025.103727)
Supplement: Supplemental Tables [file mmc1.docx]

**Supplemental Table 1:** Comparison of Case Mixture in 13 Participating Institutions.

| Site | 1 | 2 | 3 | 4 | 5 | 6 | 7 | 8 | 9 | 10 | 11 | 12 | 13 |
| --- | --- | --- | --- | --- | --- | --- | --- | --- | --- | --- | --- | --- | --- |
| Cases, n | 1,487 | 1,056 | 981 | 2,733 | 1,830 | 1,259 | 2,398 | 1,377 | 1,425 | 477 | 1,179 | 1,172 | 1,229 |
| Age, y (median) | 2.9 | 3.3 | 2.4 | 4.7 | 10.9 | 3.0 | 6.3 | 3.0 | 4.0 | 2.0 | 4.6 | 8.0 | 3.1 |
| <1 y | 540 (36%) | 364 (35%) | 361 (37%) | 733 (27%) | 442 (23%) | 445 (35%) | 614 (26%) | 467 (34%) | 439 (31%) | 206 (43%) | 400 (34%) | 250 (21%) | 402 (33%) |
| 1 – <18 y | 706 (47%) | 543 (51%) | 559 (57%) | 1,655 (60%) | 912 (50%) | 694  (55%) | 1,353 (56%) | 818 (59%) | 760  (53%) | 174 (37%) | 580 (49%) | 707 (60%) | 770 (63%) |
| >=18 y | 241 (16%) | 149 (14%) | 61 (6%) | 345  (13%) | 496 (27%) | 120 (10%) | 431 (18%) | 92 (7%) | 226 (16%) | 97 (20%) | 199 (17%) | 215 (18%) | 57 (5%) |
| Weight, kg (median) | 13.8 | 13.9 | 12.4 | 16.7 | 34.3 | 12.8 | 20.4 | 14.0 | 16.0 | 11.4 | 17.2 | 23.9 | 13.8 |
| Case type | | | | | | | | | | | | | |
| Biopsy | 1  (0%) | 1  (0%) | 213  (22%) | 483  (18%) | 321  (18%) | 0  (0%) | 720  (30%) | 226  (16%) | 36  (2.5%) | 1  (0%) | 49  (4%) | 299  (36%) | 79  (6%) |
| Diagnostic | 610  (41%) | 320 (30%) | 391  (40%) | 742  (27%) | 611  (33%) | 667  (53%) | 713  (30%) | 319  (23%) | 764  (54%) | 123  (26%) | 444  (38%) | 369  (32%) | 307  (25%) |
| Interventional | 876  (59%) | 735  (70%) | 377  (38%) | 1,508  (55%) | 898  (49%) | 592  (47%) | 965  (40%) | 832  (60%) | 625  (44%) | 353  (74%) | 686  (58%) | 504  (43%) | 843  (69%) |
| REC | | | | | | | | | | | | | |
| REC I | 1,098  (74%) | 695  (66%) | 861  (88%) | 1,959  (72%) | 1,490  (81%) | 1,025  (81%) | 1,851  (77%) | 999  (73%) | 1,198  (84%) | 323  (68%) | 854  (72%) | 1,005  (86%) | 876  (71%) |
| REC II | 290  (20%) | 253  (24%) | 81  (8%) | 426  (16%) | 253  (14%) | 210  (17%) | 373  (16%) | 300  (22%) | 137  (10%) | 89  (19%) | 204  (17%) | 121  (10%) | 275  (22%) |
| REC III | 99  (7%) | 108  (10%) | 39  (4%) | 348  (13%) | 87  (5%) | 24  (2%) | 174  (7%) | 78  (6%) | 90  (6%) | 65  (14%) | 121  (10%) | 46  (4%) | 78  (6%) |
| Median μGym2/kg | | | | | | | | | | | | | |
| REC I | 21.4 | 51.7 | 36.8 | 8.8 | 30.7 | 54.1 | 13.8 | 23.3 | 9.8 | 21.3 | 6.3 | 28.3 | 10..7 |
| REC II | 46.9 | 106.8 | 97.0 | 21.2 | 90.1 | 115.6 | 36.2 | 78.2 | 24.5 | 128.2 | 17.4 | 106 | 22.0 |
| REC III | 64.0 | 172.9 | 149.1 | 35.7 | 112.2 | 175.0 | 59.0 | 143.2 | 61.0 | 157.4 | 31.9 | 103 | 52.9 |
| Total | 27.5 | 74.1 | 42.0 | 13.0 | 38.0 | 63.3 | 17.9 | 34.2 | 11.6 | 40.3 | 9.0 | 34.8 | 14.3 |
| SAE rate | | | | | | | | | | | | | |
| REC I | 1.8% | 4.3% | 4.1% | 4.2% | 2.1% | 1.6% | 3.4% | 3.3% | 2.9% | 6.8% | 1.6% | 4.5% | 1.3% |
| REC II | 2.1% | 6.3% | 6.2% | 4.9% | 2.8% | 3.3% | 5.1% | 5.7% | 13.1% | 10.1% | 3.4% | 9.1% | 3.3% |
| REC III | 2.0% | 5.6% | 12.8% | 8.9% | 6.9% | 0% | 6.3% | 11.5% | 2.2% | 20% | 4.1% | 10.9% | 2.6% |
| Total | 1.9% | 4.9% | 4.6% | 4.9% | 2.4% | 1.8% | 3.9% | 4.3% | 3.9% | 9.2% | 2.2% | 5.2% | 1.8% |
| Sheath time (min) | | | | | | | | | | | | | |
| REC I | 54 | 49 | 74 | 61 | 69 | 72 | 55 | 49 | 107 | 101 | 59 | 70 | 55 |
| REC II | 78 | 85 | 132 | 116 | 142 | 124 | 127 | 108 | 164 | 199 | 128 | 190 | 90 |
| REC III | 93 | 126 | 184 | 150 | 161 | 192 | 183 | 171 | 196 | 274 | 189 | 193 | 125 |
| Total | 60 | 62 | 78 | 75 | 79 | 78 | 66 | 64 | 115 | 125 | 76 | 78 | 64 |
| Fluoroscopy time (min) | | | | | | | | | | | | | |
| REC I | 8 | 11 | 9 | 13 | 14 | 14 | 10 | 11 | 16 | 21 | 10 | 14 | 11 |
| REC II | 15 | 25 | 18 | 33 | 30 | 29 | 29 | 33 | 34 | 70 | 29 | 45 | 23 |
| REC III | 17 | 42 | 29 | 43 | 41 | 46 | 42 | 63 | 44 | 89 | 37 | 46 | 36 |
| Total | 10 | 16 | 14 | 17 | 16 | 16 | 13 | 15 | 18 | 31 | 14 | 16 | 14 |

**Supplemental Table 2.** Radiation Dosage Stratified by Radiation Exposure Category

|  | Radiation exposure category, μGym2/kg | | | | | | *p* value |
| --- | --- | --- | --- | --- | --- | --- | --- |
|  | Low | | Medium | | High | |  |
|  | n | Median (IQR) | n | Median (IQR) | n | Median (IQR) |  |
|  | 14,234 | 18.2 (7.1 – 44.7) | 3,012 | 49.8 (20.9 – 107.9) | 1,357 | 67.0 (30.7 – 140.7) | <0.001 |
| Study period | | | | | | | |
| 1 (1/2016 –  6/2018) | 6,830 | 20.3 (7.8 – 49.4) | 1,516 | 61.0 (25.5 – 123.5) | 610 | 80.6 (36.2 – 150.1) | <0.001 |
| 2 (7/2018 –  12/2020) | 7,404 | 16.6 (6.5 – 40.9) | 1,496 | 41.0 (18.5 – 88.2) | 747 | 56.3 (24.8 – 130.2) | <0.001 |
| Year | | | | | | | |
| 2016 | 2,444 | 21.4 (8.3 – 52.4) | 533 | 61.7 (25.8 – 130.6) | 203 | 72.6 (35.4 – 141.4) | <0.001 |
| 2017 | 2,893 | 22.4 (8.6 – 52.3) | 646 | 68.8 (30.6 – 129.0) | 261 | 89.1 (41.9 – 152.3) | <0.001 |
| 2018 | 3,105 | 17.5 (6.3 – 42.5) | 642 | 49.1 (20.5 – 103.2) | 295 | 66.2 (29.6 – 149.0) | <0.001 |
| 2019 | 3,116 | 16.2 (6.2 – 41.0) | 610 | 41.0 (17.1 – 95.0) | 285 | 54.2 (23.6 – 121.9) | <0.001 |
| 2020 | 2,676 | 16.0 (6.7 – 38.7) | 561 | 37.7 (18.0 – 75.5) | 313 | 60.1 (25.4 – 133.7) | <0.001 |
| Age group | | | | | | | |
| Age <1 yr | 4,500 | 19.4 (8.8 – 45.7) | 901 | 46.5 (18.9 – 87.4) | 242 | 56.9 (29.0 – 131.4) | <0.001 |
| Age 1 – <18y | 7,662 | 15.0 (5.5 – 35.7) | 1,836 | 47.4 (20.1 – 102.4) | 733 | 59.3 (27.4 – 124.2) | <0.001 |
| Age ≥18y | 2,072 | 35.7 (12.4 – 87.1) | 275 | 108.6 (46.8 – 191.3) | 382 | 88.2 (41.8 – 206.1) | <0.001 |
| Significant adverse event | | | | | | | |
| No | 13,796 | 17.9 (7.0 – 43.9) | 2,860 | 48.7 (20.5 – 105.4) | 1,260 | 64.1 (30.3 – 134.1) | <0.001 |
| Yes | 438 | 27.4 (12.1 – 73.8) | 152 | 84.7 (28.9 – 186.4) | 97 | 117.9 (46.5 – 266.9) | <0.001 |
| Site | | | | | | | |
| 1 | 1,098 | 21.4 (10.9 – 48.4) | 290 | 46.9 (25.0 – 77.8) | 99 | 64.0 (37.3 – 147.3) | <0.001 |
| 2 | 695 | 51.7 (25.7 – 98.4) | 253 | 106.8 (61.6 – 188.0) | 108 | 172.9 (111.2 – 262.3) | <0.001 |
| 3 | 861 | 36.8 (20.3 – 65.3) | 81 | 97.0 (58.3 – 144.6) | 39 | 149.1 (105.2 – 226.0) | <0.001 |
| 4 | 1,959 | 8.8 (3.7 – 18.9) | 426 | 21.2 (12.9 – 38.1) | 348 | 35.7 (19.5 – 62.8) | <0.001 |
| 5 | 1,490 | 30.7 (16.0 – 57.9) | 253 | 90.1 (56.1 – 130.8) | 87 | 112.2 (73.3 – 179.3) | <0.001 |
| 6 | 1,025 | 54.1 (22.7 – 108.9) | 210 | 115.6 (68.9 – 213.0) | 24 | 175.0 (5.4 – 354) | <0.001 |
| 7 | 1,851 | 13.8 (5.4 – 32.7) | 373 | 36.2 (17.8 – 75.3) | 174 | 59.0 (27.0 – 148.7) | <0.001 |
| 8 | 999 | 23.3 (9.9 – 55.1) | 300 | 78.2 (46.0 – 136.0) | 78 | 143.2 (77.0 – 251.1) | <0.001 |
| 9 | 1,198 | 9.8 (5.3 – 18.5) | 137 | 24.5 (15.7 – 48.2) | 90 | 61.0 (33.9 – 92.4) | <0.001 |
| 10 | 323 | 21.3 (10.1 – 52.6) | 89 | 128.2 (56.0 – 199.5) | 65 | 157.4 (82.4 – 295.1) | <0.001 |
| 11 | 854 | 6.3 (2.7 – 12.2) | 204 | 17.4 (10.9 – 32.6) | 121 | 31.9 (20.2 – 58.0) | <0.001 |
| 12 | 1,005 | 28.3 (9.3 – 59.4) | 121 | 106.1 (59.4 – 168.5) | 46 | 102.6 (58.4 – 208.0) | <0.001 |
| 13 | 876 | 10.7 (4.2 – 25.4) | 275 | 22.0 (10.1 – 53.1) | 78 | 52.9 (19.8 – 104.8) | <0.001 |

**Supplemental Table 3.** Comparison of patient and procedural characteristics between study period 1 (1/2016 – 6/2018) and period 2 (7/2018 – 12/2020).

|  | | | | Study period 1  1/2016 – 6/2018 | Study period 2  7/2018 – 12/2020 | P-value |
| --- | --- | --- | --- | --- | --- | --- |
|  |  |  |  | n= 8,956 | n= 9,647 |  |
| Median μGym2/kg (IQR) | | | All cases | 27.2 (9.8 – 67.5) | 21.1 (8.3 – 52.9) | <0.001 |
| Median μGym2/kg (IQR) | <18 y | | REC I (Low) | 18.4 (7.3 – 42.4) | 15.2 (6.2 – 36.3) | <0.001 |
|  |  |  | REC II (Medium) | 56.7 (23.1 – 114.6) | 38.5 (17.9 – 80.9) | <0.001 |
|  |  |  | REC III (High) | 70.1 (33.3 – 127.9) | 53.0 (22.8 – 121.7) | <0.001 |
|  | ≥18 y | | REC I (Low) | 39.6 (13.7 – 96.9) | 31.6 (11.4 – 80.0) | <0.001 |
|  |  |  | REC II (Medium) | 128.3 (53.5 – 203.6) | 95.3 (37.8 – 157.0) | 0.024 |
|  |  |  | REC III (High) | 112.9 (56.6 – 256.1) | 71.3 (32.8 – 168.6) | <0.001 |
| Patient characteristics | | | | | | |
| Age group, n | | <1 y | | 2,542 (28.4%) | 3,101 (32.1%) | <0.001 |
|  |  | 1 – <18 y | | 5,019 (56.0%) | 5,212 (54.0%) |  |
|  |  | ≥18 y | | 1,395 (15.6%) | 1,334 (13.8%) |  |
| Weight, kg, median (IQR) | | | | 17.0 (7.5 – 51.2) | 16.0 (6.6 – 49.3) | <0.001 |
| CRISP category  (<18 y), n | | 1 | | 1,561 (20.6%) | 1,568 (18.9%) | 0.061 |
|  |  | 2 | | 2,727 (36.1%) | 2,937 (35.3%) |  |
|  |  | 3 | | 2,356 (3.21%) | 2,779 (33.4%) |  |
|  |  | 4 | | 860 (11.4%) | 950 (11.4%) |  |
|  |  | 5 | | 57 (0.8%) | 78 (0.9%) |  |
| CRISA category (>=18 y), n | | 1 | | 213 (15.3%) | 185 (13.9%) | 0.377 |
|  |  | 2 | | 1,055 (75.6%) | 1,004 (75.3%) |  |
|  |  | 3 | | 113 (8.1%) | 128 (9.6%) |  |
|  |  | 4 | | 14 (1.0%) | 17 (1.3%) |  |
| Procedural characteristics | | | | | | |
| Radiation exposure categories REC, n | | REC I (Low) | | 6,830 (76.3%) | 7,404 (76.7%) | 0.003 |
|  |  | REC II (Medium) | | 1,516 (16.9%) | 1,496 (15.5%) |  |
|  |  | REC III (High) | | 610 (6.8%) | 747 (7.7%) |  |
| Case type, n | | Biopsy | | 1,183 (13.2%) | 1,246 (12.9%) | 0.014 |
|  |  | Diagnostic | | 3,155 (35.2%) | 3,225 (33.4%) |  |
|  |  | Interventional | | 4,618 (51.6%) | 5,176 (53.7%) |  |
| Sheath time, min, median (IQR) | | REC I (Low) | | 67 (45 – 97) | 62 (42 – 91) | <0.001 |
|  |  | REC II (Medium) | | 119 (86 – 165) | 115 (81 – 163) | 0.181 |
|  |  | REC III (High) | | 164 (120 – 222) | 159 (117 – 213) | 0.898 |
| Fluoroscopy time, min, median (IQR) | | REC I (Low) | | 13 (8 – 20) | 12 (7 – 19) | <0.001 |
|  |  | REC II (Medium) | | 29 (18 – 43) | 29 (18 – 44) | 0.168 |
|  |  | REC III (High) | | 43 (30 – 63) | 41 (27 – 63) | 0.272 |

**Supplemental Table 4.** Comparison of radiation exposure dosage between infants, children, and adults in certain procedure types

|  | | Total | <1 year | 1 - <18 year | >=18 year | P value |
| --- | --- | --- | --- | --- | --- | --- |
| Diagnostic catheterization | n | 5551 | 2175 | 3129 | 1076 |  |
|  | Median  DAP/kg | 22.4  (8.3-57.6) | 23.2  (10.4-54.3) | 17.7  (6.4-44.5) | 45.2  (13.4-106) | <0.001 |
| ASD or PFO closure | n | 1395 | 53 | 991 | 351 |  |
|  | Median  DAP/kg | 11.6  (4.6-25.9) | 13.1  (5.5-31.1) | 9.0  (3.5-189) | 22.6  (11.0-47.9) | <0.001 |
| Proximal pulmonary angioplasty or stent | n | 834 | 211 | 562 | 61 |  |
|  | Median  DAP/kg | 47.5  (17.9-99.9) | 49.5  (18.0-105) | 40.2  (16.3-92.3) | 99.8  (53.2-200) | <0.001 |
| TPV implantation | n | 542 | NA | 248 | 294 |  |
|  | Median  DAP/kg | 68.6  (31.8-163) | NA | 53.6  (27.7-129) | 79.7  (35.8-201) | 0.002 |

**Supplemental Table 5.** Yearly trend of median sheath and fluoroscopy time stratified by radiation exposure category (REC)

|  |  | 2016 | 2017 | 2018 | 2019 | 2020 |
| --- | --- | --- | --- | --- | --- | --- |
| REC I  (Low) | Sheath time, min | 67  (44-98) | 67  (45-97) | 64  (44-96) | 62  (41-92) | 61  (41-89) |
|  | Fluoro time, min | 13  (7-20) | 13  (8-20) | 12  (8-20) | 12  (7-19) | 12  (7-19) |
| REC II (Medium) | Sheath time, min | 117  (83-158) | 120  (88-169) | 120  (87-165) | 113  (78-159) | 116  (79-215) |
|  | Fluoro time, min | 27  (18-40) | 31  (19-47) | 29  (18-44) | 29  (18-45) | 28  (18-43) |
| REC III (High) | Sheath time, min | 159  (117-207) | 168  (121-231) | 172  (119-225) | 156  (111-215) | 158  (119-203) |
|  | Fluoro time, min | 40  (28-59) | 43  (30-64) | 43  (28-64) | 39  (26-59) | 43  (28-65) |

**Supplemental Table 6.** Radiation Reduction Practice Questionnaire Results

| Question | Answer |
| --- | --- |
| Did your center have an active radiation reduction QI initiative during this time frame (Jan 2016- Dec 2020)? | Yes (3, 23.1%), No (10, 76.9%) |
| What is your default "Fluoroscopy pulse/frame rate? | 2-3 fps (3, 23.1%), 4-6 fps (3, 23.1%), 7-8 fps (5, 38.5%), 8-15 fps (2, 15.4%), 15-30 fps (0, 0.0%) |
| Was there a change in the default pulse/frame rate (fluoroscopy) over the study time frame (Jan 2016- Dec 2020)? | Yes (2, 15.4%), No (11, 84.6%) |
| What is your default cine (digital angiography) frame rate? | 7-8 fps (1, 7.7%), 15 fps (11, 84.6%), 30 fps (1, 7.7%) |
| Was there a change in the default pulse/frame rate for cine/digital angiography over the study time frame (Jan 2016- Dec 2020)? | Yes (2, 15.4%), No (11, 84.6%) |
| Does your team include a discussion of the frame rates before/during the catheterization and make changes for each patient (as part of brief/timeout)? | Yes (6, 46.2%), No (7, 53.8%) |
| Was there a replacement/upgrade of the catheterization lab angiographic system during this study time frame (Jan 2016- Dec 2020)? If yes, year (institutions) | Yes (6, 46.2%). 2016 (1), 2018 (2), 2019 (2), 2020 (1) |
| Does your program have fellows or trainees in the cardiac catheterization laboratory? | Yes (11, 84.6%), No (2, 15.4%) |
| Does your center use special angiographic imaging that may affect radiation? | 3D-Rotational angiography (3DRA) (8, 61.5%), Digital subtraction angiography for certain cases (3, 23.1%), CT/MRI/echo overlay (5, 38.5%), None (5, 38.5%) |
| Do you believe special angiographic imaging (3DRA, DSA, CTA/CMR/echocardiogram) overlay have affected your radiation doses? | Yes (4, 50.0%), No (4, 50.0%) |
| Do you use a single plane even when a biplane is available for certain procedures and interventions such as ASD closure, coarctation stenting, or other? And if so, how often? | Yes (8, 61.5%)  Rarely (< 1%) (1, 12.5%), Occasionally (1-5%) (5, 62.5%), Often (>5%) (2, 25.0%) |
| Do you have an institutional policy to follow up with patients with high radiation exposure for nonstochastic effects such as skin erythema/injury, radiation sickness, etc? What is the cut-off used (institutions) | Yes (9, 69.2%).  2000 mGy (3), 3000 mGy (2), 5000 mGy (1), and 6000 mGy (1), 2 institutions use fluoroscopy time |
| Fps frame per second. mGy milligray. 3DRA 3D rotational angiography. DSA Digital substraction angiography, CTA Cardiac computer tomography angiogram, CMR Cardiac magnetic resonance | |
